# MATRICS: A Method for Aggregating The Reporting of Interventions in Complex Studies

**DOI:** 10.1186/1745-6215-12-S1-A147

**Published:** 2011-12-13

**Authors:** Kymberley Thorne, Gabi S Jerzembek, Wai-Yee Cheung, David Cohen, Hayley A Hutchings, Frances L Rapport, Anne C Seagrove, John G Williams, Ian T Russell

**Affiliations:** 1 Centre for Health Information Research and Evaluation, Grove Building, College of Medicine, Swansea University, Singleton Park, Swansea, SA2 8PP, UK; 2 Institute of Health Service Effectiveness (IHSE), Aston University Business School, Aston Triangle, Birmingham, B4 7ET, UK; 3 Faculty of Health Sport and Science, University of Glamorgan, Pontypridd, South Glamorgan CF37 1DL, UK

## Background

There are few rigorous methods for combining qualitative and quantitative findings from studies with complex interventions using multiple research methods and giving appropriate weight to each without introducing bias to the overall conclusions.

We developed a *Method for Aggregating The Reporting of Interventions in Complex Studies* (*MATRICS*) for the ENIGMA study (Evaluating Innovations in Gastroenterology by the NHS Modernisation Agency) – a multi-centre, mixed-methods study to evaluate the impact of the Modernising Endoscopy Services programme [1], funded by the UK National Institute for Health Research (NIHR SDO ref 08/1304/46).

## Method

We developed a template that requires researchers to follow the steps outlined below:

1. List the types of effects identified by the study (from the aims objectives and outcome measures), and divide them between effects on: sample population (eg patients, carers); on the specialty being investigated (eg intensive care, outpatients) and on the rest of the organisation and society and give each a unique number.

2. List the methods used to explore each effect listed in step 1 and give each a unique letter (eg GP interviews, patient questionnaires, routine data linkage).

3. Create an alphanumeric code by cross matching the effects identified and the methods used to investigate them (eg patient satisfaction “1” was investigated using a patient questionnaire “A” = 1A).

4. List the explicit findings of the study and label them using the alphanumeric code (eg “patients were dissatisfied with waiting times – A1”).

5. Synthesise all consistent findings and list their alphanumeric codes alongside to characterise mutually confirmatory findings. Synthesis is best done independently by at least two researchers.

6. Reorder all contradictory findings and their alphanumeric codes adjacent to one another to better illustrate all conflicting findings.

## Findings

The MATRICS tool greatly facilitated the unbiased factual reporting of findings from multiple methods for ENIGMA [1]. Additionally, it was most beneficial for the qualitative synthesis of the findings of ENIGMA, a study unsuited to formal cost-benefit analysis, like most in this field.

We have also applied the MATRICS successfully to other complex studies using multiple methods.

## Discussion

If the experience of the study team regarding the MATRICS approach to synthesising results in complex studies is reflected by others, it could provide a formal structure for reporting the results of complex and/or multiple-method studies. Further application of this methodology will provide evidence of whether this reporting tool will improve a reader’s understanding of a study and its findings.
